# Supplementary material for: Temporo-parietal cortex involved in modeling one’s own and others’ attention
Source: eLife. 2021 Feb 15;10:e63551. doi: 10.7554/eLife.63551 (PMC7884070; doi:10.7554/eLife.63551)
Supplement: Supplementary file 1. — All clusters (≥10 voxels) of decoding activity passing the voxelwise threshold of p<0.001 (none of the clusters survived p<0.05 correction for multiple comparisons using the whole brain as search space). *The left posterior STS cluster also coincided with the posterior TPJ (TPJp) subregion as defined by Bzdok et al., 2013; Mars et al., 2012. [file elife-63551-supp1.docx]

| **Anatomical region** | **Peak MNI** | **Peak t** | **Cluster size** |
| --- | --- | --- | --- |
| L. posterior STS (TPJ*) | -59, -47, 5 | 4.21 | 22 |
| R. middle frontal gyrus | 39, -7, 55 | 4.15 | 17 |
| R. inferior precentral sulcus | 42, 18, 15 | 4.10 | 37 |
| L. lingual gyrus | -11, -80, -8 | 3.97 | 11 |

**Supplementary File 1. Decoding endogenous versus exogenous at the whole-brain level**. All clusters (≥10 voxels) of decoding activity passing the voxelwise threshold of p < 0.001 (none of the clusters survived p < 0.05 correction for multiple comparisons using the whole brain as search space). *The left posterior STS cluster also coincided with the posterior TPJ (TPJp) subregion as defined by (Bzdok et al., 2013; Mars et al., 2012).
