# Supplementary material for: Temporo-parietal cortex involved in modeling one’s own and others’ attention
Source: eLife. 2021 Feb 15;10:e63551. doi: 10.7554/eLife.63551 (PMC7884070; doi:10.7554/eLife.63551)
Supplement: Supplementary file 2. — All clusters (≥10 voxels) decoding agent type (self versus other) activity at the threshold of p<0.001 (uncorrected). Corrected p values represent cluster-level correction using the whole brain as search space. [file elife-63551-supp2.docx]

| **Anatomical region** | **Peak MNI** | **Peak t** | **Cluster size** | **Corrected p value** |
| --- | --- | --- | --- | --- |
| L. angular gyrus (TPJ) | -59, -67, 27 | 5.92 | 211 | <0.001 |
| R. precuneus | 4, -55, 30 | 5.91 | 57 | - |
| L. inferior temporal gyrus | -41, -15, -18 | 5.49 | 131 | 0.004 |
| L. inferior occipital gyrus | -31, -77, -8 | 5.26 | 54 | - |
| R. middle cingulate cortex | 2, -15, 40 | 4.24 | 47 | - |
| R. lingual gyrus | 24, -82, -8 | 4.82 | 121 | 0.006 |
| L. fusiform gyrus | -39, -57, -23 | 4.81 | 114 | 0.008 |
| R. calcarine sulcus | 7, -85, 5 | 4.71 | 53 | - |
| R. fusiform gyrus | 27, -37, -21 | 4.32 | 34 | - |
| R. inferior occipital gyrus | 47, -80, 3 | 4.05 | 27 | - |
| L. inferior frontal sulcus | -41, 13, 27 | 4.02 | 13 | - |
| L. superior frontal gyrus | -29, 16, 65 | 3.98 | 12 | - |

**Supplementary File 2. Decoding agent type at the whole-brain level**. All clusters (≥10 voxels) decoding agent type (self versus other) activity at the threshold of p < 0.001 (uncorrected). Corrected p values represent cluster-level correction using the whole brain as search space.
