# Supplementary material for: Temporo-parietal cortex involved in modeling one’s own and others’ attention
Source: eLife. 2021 Feb 15;10:e63551. doi: 10.7554/eLife.63551 (PMC7884070; doi:10.7554/eLife.63551)
Supplement: Supplementary file 3. — All clusters (≥10 voxels) in which endogenous-versus-exogenous decoding was better in self-related compared to other-related stories at p<0.001 (uncorrected). (none of the clusters survived correction for multiple comparisons using the whole brain as search space). [file elife-63551-supp3.docx]

| **Anatomical region** | **Peak MNI** | **Peak t** | **Cluster size** |
| --- | --- | --- | --- |
| L. posterior STS | -51, -27, -1 | 4.20 | 13 |
| R. inferior temporal gyrus | 47, -22, -28 | 3.96 | 13 |

**Supplementary File 3. Decoding attention-by-agent interaction at the whole-brain level**. All clusters (≥10 voxels) in which endogenous-versus-exogenous decoding was better in self-related compared to other-related stories at p < 0.001 (uncorrected). (none of the clusters survived correction for multiple comparisons using the whole brain as search space).
