# Supplementary material for: Temporo-parietal cortex involved in modeling one’s own and others’ attention
Source: eLife. 2021 Feb 15;10:e63551. doi: 10.7554/eLife.63551 (PMC7884070; doi:10.7554/eLife.63551)
Supplement: Supplementary file 4. — Clusters (≥10 voxels) decoding social versus nonsocial stories significantly better than chance. The listed clusters represent the overlap of significant clusters (p<0.05, corrected using a cluster-defining uncorrected threshold of p<0.001 and the entire brain as search space) across four separate whole-brain searchlight analyses: endogenous-self versus nonsocial, exogenous-self versus nonsocial, endogenous-other versus nonsocial, and exogenous-other versus nonsocial. [file elife-63551-supp4.docx]

| **Anatomical region** | **Cluster size** |
| --- | --- |
| L. TPJ | 1722 |
| L. and R. precuneus | 80 |
| R. intraparietal sulcus | 137 |
| L. inferior frontal sulcus | 22 |

**Supplementary File 4. Decoding social versus nonsocial stories at the whole-brain level**. Clusters (≥10 voxels) decoding social versus nonsocial stories significantly better than chance. The listed clusters represent the overlap of significant clusters (p < 0.05, corrected using a cluster-defining uncorrected threshold of p < 0.001 and the entire brain as search space) across four separate whole-brain searchlight analyses: endogenous-self versus nonsocial, exogenous-self versus nonsocial, endogenous-other versus nonsocial, and exogenous-other versus nonsocial.
