# Supplementary material for: Temporo-parietal cortex involved in modeling one’s own and others’ attention
Source: eLife. 2021 Feb 15;10:e63551. doi: 10.7554/eLife.63551 (PMC7884070; doi:10.7554/eLife.63551)
Supplement: Supplementary file 5. — All clusters (k ≥ 10) of voxels surviving a p<0.001 (uncorrected) threshold are shown for the univariate contrasts corresponding to the main multivariate analyses (Figures 3–4). The preprocessed functional images were smoothed using a 6 mm full FWHM Gaussian kernel and subjected to conventional GLMs. We report the p values for clusters that survived a cluster-level familywise error rate correction for multiple comparisons, either using the whole brain or one of the ROI volumes as search space. [file elife-63551-supp5.docx]

|  | **Peak MNI** | | **Peak t** | **Cluster size** | **P value (whole-brain corr)** | **P value (corr within ROI)** |
| --- | --- | --- | --- | --- | --- | --- |
| ***ENDO > EXO*** | | | | | |  |
| No significant voxels. |  |  | |  |  |  |
| ***EXO > ENDO*** |  |  | |  |  |  |
| No significant voxels. |  |  | |  |  |  |
| **SELF > OTHER** |  |  | |  |  |  |
| No significant voxels. |  |  | |  |  |  |
| **OTHER > SELF** |  |  | |  |  |  |
| L. calcarine sulcus | -14, -87, 5 | 6.53 | | 497 | <0.001 | - |
| ***(ENDO-SELF > EXO-SELF) > (ENDO-OTHER > EXO-OTHER)*** | | | | | | |
| R. rectal gyrus | 2, 53, -23 | 4.57 | | 13 | - | - |
| L. posterior IPS | -29, -87, 40 | 3.94 | | 11 | - | - |
| R. middle frontal gyrus | 49, 26, 40 | 3.94 | | 19 | - | - |
| R. parahippocampal gyrus | 22, -5, -28 | 3.82 | | 13 | - | - |
| L. calcarine gyrus | -11, -72, 20 | 3.71 | | 11 | - | - |
| L. parietooccipital sulcus | -49, 28, -3 | 3.65 | | 10 | - | - |
| ***(EXO-SELF > ENDO-SELF) > (EXO-OTHER > ENDO-OTHER)*** | | | | | | |
| No significant voxels. |  |  | |  |  |  |
| ***Mean(ALL SOCIAL CONDITIONS) > NONSOCIAL CONTROL*** | | | | | | |
| L. middle orbital gyrus | -9, 58, -8 | 5.23 | | 429 | <0.001 | - |
| R. supramarginal gyrus | 59, -30, 35 | 4.72 | | 214 | 0.002 | - |
| L. superior frontal gyrus | -16, 38, 35 | 4.71 | | 222 | 0.002 | - |
| L. mid-insula | -39, 3, 2 | 4.56 | | 122 | 0.029 | - |
| L. cuneus | -6, -92, 32 | 4.35 | | 11 | - | - |
| R. operculum | 44, 8, 10 | 4.31 | | 37 | - | - |
| R. superior medial gyrus | 9, 63, 10 | 4.27 | | 17 | - | - |
| R. postcentral gyrus | 27, -47, 57 | 4.04 | | 12 | - | - |
| L. supramarginal gyrus | -56, -27, 22 | 3.96 | | 19 | - | - |

**Supplementary File 5. Univariate fMRI results.** All clusters (k≥10) of voxels surviving a p<0.001 (uncorrected) threshold are shown for the univariate contrasts corresponding to the main multivariate analyses (Figures 3-4). The preprocessed functional images were smoothed using a 6-mm full FWHM Gaussian kernel and subjected to conventional GLMs. We report the p values for clusters that survived a cluster-level familywise error rate correction for multiple comparisons, either using the whole brain or one of the ROI volumes as search space.
