# Supplementary material for: Temporo-parietal cortex involved in modeling one’s own and others’ attention
Source: eLife. 2021 Feb 15;10:e63551. doi: 10.7554/eLife.63551 (PMC7884070; doi:10.7554/eLife.63551)
Supplement: Supplementary file 7. [file elife-63551-supp7.docx]

**Supplementary File 7**

**Searchlight Analysis**

Our primary analysis, described in the main text, was a targeted testing of strong hypotheses within a set of defined RIOs. Such targeted testing is preferred because it puts strong hypotheses to a direct test, and because it is more sensitive, avoiding the statistical problems of a brain-wide multiple comparison correction. Given the extremely subtle differences between the story types in the present experiment, such a targeted and sensitive analysis was preferred. However, in addition to the targeted ROI analyses, we also performed an exploratory analysis to ask whether any meaningful decoding activity might be identified outside of the ROIs. We used a searchlight analysis (Kriegeskorte et al., 2006). The searchlight analysis is fundamentally different from the ROI analysis. It is not targeted to specific brain areas on the basis of predictions. Instead, it is a whole-brain analysis that is much more statistically conservative because of the brain-wide multiple comparisons. In general, one would not expect the searchlight analysis to align perfectly with the ROI analysis. Activations revealed in the more sensitive ROI analysis might not appear in the searchlight analysis. Instead, it is exploratory in nature, and its usefulness is that it may reveal clusters of strong decoding in unanticipated areas outside the ROIs.

*Endogenous-vs-exogenous searchlight analysis*

First, the brain was partitioned into overlapping voxel clusters of spherical shape (10-mm radius). In each of these clusters, a decoding accuracy was computed using the same model input, SVM parameters, and procedures as described for the ROI analysis. This process resulted in an endogenous-versus-exogenous decoding accuracy map for each subject, in which the value of each voxel represents the average proportion of correctly classified trials relative to chance level (50%) based on the 10 mm sphere of tissue surrounding that voxel. The subject-wise decoding maps were then smoothed using a 3-mm full-width-half-maximum (FWHM) Gaussian kernel, and entered into a second-level analysis using SPM12. At the second-level, the whole-brain decoding maps were thresholded at p < 0.001 (uncorrected for multiple comparisons). For statistical inference, we employed a cluster-level, whole-brain approach to find clusters that passed the threshold of p < 0.05, corrected for brain-wide multiple comparisons using the familywise error rate correction as implemented by SPM12. In a purely descriptive manner, we also report strong decoding activity, defined as clusters ≥ 10 voxels using the cluster-forming threshold of p < 0.001 uncorrected, that did not survive correction at the whole-brain level (Supplementary File 1).

Clusters revealed in the searchlight analysis were projected onto orthogonal sections of the average structural scan generated from the 32 subjects for anatomical localization. The decoding clusters were also projected onto a 3D canonical brain surface using the software Surf Ice (University of South Carolina, McCausland Center for Brain Imaging). Figure 3-figure supplement 3 shows the brain-wide peak in decoding obtained with the searchlight method.

The searchlight analysis revealed no clusters that were brain-wide significant at the corrected p < 0.05 threshold. When examining the voxel-wise p < 0.001 threshold, four clusters (≥10 voxels) were observed (see Supplementary File 1 for details). The strongest peak decoding (t = 4.21) was located in the left posterior STS (see Figure 3-figure supplement 3). Although this peak was located 22 mm anterior to the center of our left TPJ ROI, it does fall within the same subregion of TPJ (the posterior TPJ, TPJp) identified by several previous accounts (Bzdok et al., 2013; Mars et al., 2012), and is thus roughly consistent with our main, ROI analysis.

*Self-versus-other searchlight analysis*

The same method used for the exogenous-versus-endogenous searchlight analysis was used for the self-versus-other searchlight analysis. The analysis revealed four clusters that significantly decoded the self-versus-other distinction (p < 0.05) after correcting for multiple comparisons using the whole brain as search space. The global decoding peak was located on the left angular gyrus (part of TPJ; see Figure 3-figure supplement 4) (t = 5.92), which is compatible with our main ROI decoding results. All decoding clusters, including those passing the uncorrected threshold p < 0.001, are listed in Supplementary File 2.

*Endogenous-versus-exogenous X self-versus-other searchlight analysis*

The same method as in the previous sections was used for the interaction, or exogenous-versus-endogenous X self-versus-other, searchlight analysis. The analysis revealed no clusters that decoded the endogenous-versus-exogenous distinction significantly (p < 0.05, after correcting for multiple comparisons using the whole brain as search space) different in self-related compared to other-related stories. When examining the voxel-wise p < 0.001 threshold, two clusters were observed (see Supplementary File 3 for details). The strongest peak decoding (t = 4.20) was located in the left posterior STS (Figure 3-figure supplement 5). All decoding clusters, including those passing the uncorrected threshold p < 0.001, are listed in Supplementary File 3.

*Social-versus-nonsocial searchlight analysis*

For this analysis, we performed four separate whole-brain searchlight analyses, testing for decoding that distinguished social from nonsocial stories. Each of the four analyses was restricted to comparing a specific type of social story to the nonsocial control: endogenous-self versus nonsocial, exogenous-self versus nonsocial, endogenous-other versus nonsocial, and exogenous-other versus nonsocial. We first identified clusters that passed a threshold of p < 0.05 corrected using the entire brain as search space. We then found brain areas of overlap, that were ≥10 voxels in size, between the decoding clusters obtained in the four different analyses. These areas of overlap represent brain regions that showed significant decoding for the social-versus-nonsocial comparison, in a consistent manner, across all types of social stimuli. Using this method, four brain areas were obtained (see Supplementary File 4 and Figure 4-figure supplement 1 for details).

**Eye-Tracking Decoding**

To examine whether eye movement dynamics could explain our fMRI decoding results, we systematically organized eye position and saccade data into gaze pattern vectors, and submitted it to a decoding model analogue to the one used for the fMRI data (see Methods for details). Out of 32 subjects, 27 had usable eye tracking data available (five subjects had data with unacceptable levels of noise due to either the presence of glasses or a partially occluded pupil). The results showed that this classifier, based solely on eye tracking data, could not decode attention type (endogenous-versus-exogenous decoding accuracy 55.2%, p=0.102) or agent (self-versus-other decoding accuracy 52.4%, p=0.376) significantly better than chance level. See Figure 3-figure supplement 8.
